# P-977. Navigating the Tide with ACORN-HAI-LITE: Frontline Insights for Optimising Antimicrobial Surveillance Efforts in Low to Middle Income Countries

**DOI:** 10.1093/ofid/ofaf695.1176

**Published:** 2026-01-11

**Authors:** Megan Precious Kang, Wilson Goh, Yin Mo, Yang Cao

**Affiliations:** Yong Loo Lin School of Medicine, National University of Singapore, Singapore, Singapore; National University Hospital Singapore, SINGAPORE, Singapore; National University Hospital, Singapore, Not Applicable, Singapore; Singapore Clinical Research Institute, Singapore, Not Applicable, Singapore

## Abstract

**Background:**

Antimicrobial resistance (AMR) is a critical global health threat requiring strong antimicrobial surveillance (AMS) programs. Low and middle-income countries (LMICs) face unique implementation challenges due to limited resources and weak infrastructure.

**Methods:**

Our study explored frontline healthcare professionals' experiences in LMICs through a mixed-method approach (survey with 110 respondents, structured interview with 9 interviewees) to pinpoint ongoing barriers to AMR/AMS implementation and assess ACORN-HAI/II's impact. We evaluated their knowledge, attitudes, practices, implementation hurdles, and countermeasure effectiveness.

**Results:**

Our study highlights several critical interconnected issues. Limited funding, cited by two-thirds of respondents, constraints essential infrastructure, training initiatives, and data management. Furthermore, 17-25% of healthcare professionals demonstrate gaps in awareness and knowledge, impacting prescribing behaviors. One-third of respondents reported a divergence between guidelines and real-world clinical application, alongside challenges in communication and collaboration among relevant stakeholders. While ACORN-HAI/II was acknowledged for providing crucial funding and raising awareness, significant gaps remain, particularly in sustained financial support and consistent guideline adherence.

**Conclusion:**

To enhance AMR/AMS efforts in resource-limited settings, we propose strategic resource allocation through diversified funding mechanisms and prioritized investment in laboratory infrastructure and skilled personnel. Fostering a culture of responsible antibiotic usage requires empowering departmental champions to lead educational initiatives and implementing stricter regulations on antibiotic dispensing. Furthermore, transitioning from reactive to proactive strategies necessitates building trust in data, developing user-friendly data dashboards, governmental engagement and rewarding data-driven improvements in clinical practice.

By addressing these multifaceted challenges and leveraging frontline insights, we can optimize resource allocation and strengthen the long-term sustainability of effective AMR surveillance in LMICs.

**Disclosures:**

All Authors: No reported disclosures

